# Physician Anxiety and Burnout: Symptom Correlates and a Prospective Pilot Study of App-Delivered Mindfulness Training

**DOI:** 10.2196/15608

**Published:** 2020-04-01

**Authors:** Alexandra Roy, Susan Druker, Elizabeth A Hoge, Judson A Brewer

**Affiliations:** 1 Brown University Providence, RI United States; 2 University of Massachusetts Medical School Worcester, MA United States; 3 Georgetown University Washington, DC United States

**Keywords:** anxiety, burnout, mindfulness, app, mHealth, physician, smartphone, digital therapeutics

## Abstract

**Background:**

Physician burnout is on the rise, yet little is known about its relationship to anxiety. Mindfulness-based stress reduction has demonstrated decreases in anxiety, yet physicians have reported reluctance to engage in it due to significant time commitments.

**Objective:**

The aims of this study are to assess whether app-based mindfulness training can reduce anxiety in physicians and to explore if anxiety and burnout are correlated, thus leading to a reduction in both anxiety and burnout.

**Methods:**

This was a nonrandomized pilot study comprised of 34 physicians who worked in a large US health care network and reported having anxiety. The intervention was an app-based mindfulness program. The main outcome measure was anxiety, measured by the Generalized Anxiety Disorder-7 (GAD-7). The secondary outcome measures assessed burnout: cynicism and emotional exhaustion items from the Maslach Burnout Inventory.

**Results:**

GAD-7 scores decreased significantly at posttreatment (1 month after treatment initiation, 48% reduction, *P*<.001) and at the 3-month follow-up (57% reduction, *P*<.001). There was a significant correlation between anxiety and burnout (cynicism: *r*=.43; *P*=.01; emotional exhaustion: *r*=.71; *P*<.001). There was also a significant decrease in cynicism (50% reduction, *P*=.003 at posttreatment; 50% reduction, *P*=.009 at follow-up) and emotional exhaustion at both time points (20% reduction, *P*<.001 at posttreatment; 20% reduction, *P*=.003 at follow-up).

**Conclusions:**

This pilot study is the first to test an app-based mindfulness training program targeted at reducing anxiety with physicians and to demonstrate that in physicians, anxiety is correlated with burnout. These findings suggest that this may be an effective tool to reduce anxiety and burnout in physicians.

**Trial Registration:**

ClinicalTrials.gov NCT04137081; https://www.clinicaltrials.gov/ct2/show/NCT04137081

## Introduction

Physician burnout has gained increasing attention and concern over the past several years due to its effects on physicians, the direct impact it has on patient care, and the increase in prevalence. A 2011 study found that 38% of physicians reported significant symptoms of burnout *on a weekly basis,* which is 10% higher than the general working population in the United States [1]. By 2014, burnout had increased to 48% among physicians, nearly double that of the general working population, which had not shown increases in the same time period [2].

Symptoms of physician burnout include exhaustion, irritability, inability to concentrate, and cynicism, among other symptoms [3,4]. Burnout has been reported to be associated with sleep disturbances, marital difficulties, depression, and anxiety [4]. Of note, while intuitive at face value, reported links between burnout and anxiety have largely been anecdotal. Burnout and anxiety may share similar presenting symptoms such as sleep disturbance and irritability; however, to date, no studies have directly assessed the correlation between these variables in physicians.

Both institutional and individual factors are theorized to contribute to the significant increase in physician burnout over the past few years. Institutional contributors include an increase in patient loads, the introduction of electronic medical record systems, and an emphasis on cost management, among other factors [5,6]. Additionally, as the health care landscape has moved increasingly to larger corporate structures, physicians have found themselves progressively removed from their practices’ decision-making processes, including decisions pertaining to the length of patient visits and treatment approaches [7]. This trend in burnout seems to be gaining momentum despite long-standing knowledge of key related and possible causal factors; a study of physicians practicing in two Kaiser Permanente regions in the 1990s found that the single most important predictor of physician burnout was a lack of “perceived control over the practice environment” [8]. This reduced sense of control has been associated with a tendency to perceive clinical ineffectiveness and “give up more easily” [4,7].

Interestingly, the link between a perceived lack of control and tendency to give up has been extensively studied in cognitive neuroscience contexts, aptly termed “learned helplessness” (for a review, see Maier et al) [9]. Ironically, for physicians, this learned behavior may begin in medical school (or earlier), directly contributing to the development (or worsening) of anxiety and predisposing them to burnout [10]. In particular, medical students are subject to environments and situations that trigger stress and anxiety responses that can be reinforced over time through well-described operant conditioning learning pathways (reinforcement learning, positive and negative reinforcement) that can lead to feelings of helplessness and anxiety. Necessary components for this type of learning include a trigger, behavior, and reward [11,12]. Positive reinforcement learning might happen when a medical student is asked a question during hospital rounds (historically referred to as a *pimping* – trigger); if she provides the correct response (behavior), she may be rewarded with praise (reward). If she replies incorrectly, she may learn through negative reinforcement by being encouraged to look up the answer, or castigated in front of the team. Both positive and negative reinforcement can perpetuate stress and anxiety as students compete to have “the best” answers and avoid humiliation, self-imposed or otherwise.

**Figure 1:**
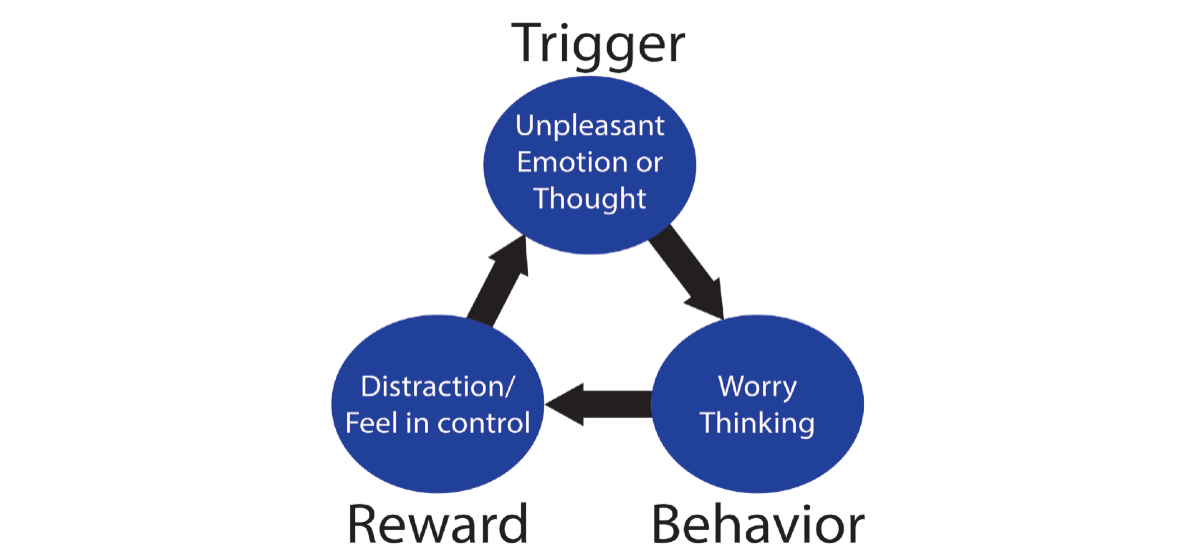
Development of a "habit loop" via positive and negative reinforcement [15,18,19].

On top of environmental causes and conditions, the high demands and inherent uncertainty in the practice of medicine may contribute to excessive worry, a core aspect of anxiety disorders [13]. From a psychological standpoint, worry represents an attempt to engage in mental problem solving for an issue with an uncertain outcome [14,15]. It has been suggested that a feeling of control over a situation can lead to the formation of a “habit loop” in which worry can be reinforced and perpetuated (ie, be positively reinforced, see Figure 1); though ironically, in this modern landscape where physician autonomy and shared decision-making are waning, this habit loop may compound anxiety and burnout [16-19]. The many pressures and institutional factors that contribute to burnout need to be addressed by health care systems; however, in the meantime, it may be possible to provide some tangible support to physicians.

Mindfulness training (MT) is gaining evidence and interest as a potential treatment for anxiety [20-25]. Mindfulness can be defined as the awareness that arises when paying attention in the present moment, on purpose and nonjudgmentally [26]. Through helping individuals observe emotions and bodily sensations instead of getting caught up in anxiety, MT has been theorized to directly target key reinforcement learning pathways [18,27]. Specifically, mindfulness helps individuals learn to identify perseverative worry thought patterns that reinforce anxiety habit loops and, importantly, to notice thoughts and emotions as mental and physical events and sensations instead of propagating the cycle. In other words, this objective observation decreases the degree to which individuals are identified with thoughts and emotions, effectively deconditioning or extinguishing the reinforcement learning process that perpetuates anxiety.

It is also unclear whether anxiety predisposes physicians to burnout or is exacerbated by burnout, or if the two interact to feed off of each other. Several studies have found that MT specifically decreases burnout in physicians, though whether components of burnout are mediated through a reduction of anxiety or another mechanism remains unknown [28-30]. Regardless, these promising findings are suggestive that MT may effectively address at least some components of burnout; however, multi-month programs such as mindfulness-based stress reduction (MBSR) are time-intensive and difficult for many physicians to incorporate into their already busy schedules. For example, in one study, 44% of health care professionals randomized to an MBSR intervention could not complete the program due to a “lack of time” [31]. This suggests a need for targeted mindfulness-based interventions that address anxiety yet are tailored to fit within physicians’ already busy lives.

App-based smartphone interventions are increasingly used to deliver behavioral treatments because of their relative low cost, high fidelity, and broad availability. This new class of “digital therapeutics” may also be an effective way to deliver MT to physicians. For example, didactic training can be tailored to be delivered in short, daily modules (eg, 10 minutes/day) rather than weekly 2-hour sessions; brief, just-in-time mindfulness tools can be accessed on demand and in context when triggered by stress or anxiety. With these factors in mind, we developed an app-based MT program for anxiety that targets the underlying reinforcement learning pathways where anxiety develops and is perpetuated. From a path model perspective, MT could reduce anxiety via a reduction in worry or an increase in nonreactivity.

Previously, we used the Obesity-Related Behavioral Intervention Trials framework to develop an app to target anxiety as a behavioral risk factor of interest (Phase I) [32]. This study (Phase II) is the next logical step to perform a “preliminary, proof-of-concept test [in] a cost-effective way to determine if a treatment package can achieve benefit on a clinically significant target in a small, select sample.”

The primary goal of this nonrandomized pilot study was to examine whether a mechanistically-based MT program delivered via app would reduce anxiety in physicians. In addition, the following questions were explored: are anxiety and burnout in physicians correlated, and can an app-based MT reduce physician burnout [33]? The primary hypothesis of this study was that app-based MT would decrease anxiety, and the secondary hypotheses were that the anxiety would be correlated with burnout and that burnout would also be reduced.

## Methods

### Participants

Physicians in the University of Massachusetts Memorial Health Care system (N=1100) were invited via email to a 30-day app-based MT program, Unwinding Anxiety (UA), geared toward anxiety. Inclusion criteria included: direct patient interaction; currently employed as a physician; owned a smartphone; answered yes to the following questions: do you feel nervous, anxious, or on edge, and do you feel you worry too much about different things?; endorsed willingness to use a mindfulness smartphone app for 10 minutes per day for 30 days; endorsed willingness to complete two surveys at 1 month and 3 months. There were no explicit exclusion criteria. Consenting participants were provided the UA app and received a $25 Amazon gift card for completion of each follow-up survey ($50 total). A control condition such as treat-as-usual was not used due to concerns about participant retention. The study was approved by the University of Massachusetts Medical School Institutional Review Board.

### Intervention

The app-based MT program teaches individuals to understand how anxious worry is developed and perpetuated through reinforcement learning, how to recognize these anxiety habit loops, and how to bring mindful awareness to moments of stress and worry, so they can uncouple feelings of anxiety from reactive worry thinking and mindfully work with habitual mind states that perpetuate and reinforce anxiety. This process helps individuals “unlearn” or extinguish worry at a core mechanistic level. This experiential education is delivered via a smartphone-based platform**,** which includes a progression of more than 30 daily modules of brief didactic and experience-based MT (videos and animations, approximately 10 min/day, see Textbox 1), app-triggered check-ins, user-initiated guided meditations (5-15 minutes), and brief (30 seconds) on-demand mindfulness exercises to help disrupt anxiety cycles in vivo. The content for this intervention was developed based upon a combination of clinical experience for individuals with anxiety and previously developed in-person and app-based MT protocols for habit change that have yielded clinically-meaningful outcomes such as cessation of smoking or overeating [18,34-36].

**Overview of Unwinding Anxiety themes and content.**

**Modules 1-7: goals; curiosity; reinforcement learning; body scan; self-monitoring**

Sets goals and introduces how habits are formed around worry (eg, reinforcement learning, distraction). Introduces curiosity to foster the nonjudgmental aspects of mindfulness and basic mindfulness practices including the body scan. Unpacks worry and fear both from a brain and behavior perspective**.**

**Modules 8-14: noting practice; RAIN; barriers to change; reinforce concepts**

Introduces how to mindfully work with worry cues and affective states using RAIN (Recognize, Accept, Investigate, and Note what emotions feel like as they arise and pass away) [34,37]. These also build on basic mindfulness using noting practice (the N of RAIN) during everyday life, and introduce additional animations to reinforce mindfulness concepts that show how we feed our anxiety by worry thinking and distraction.

**Modules 15-21: noting practice (cont’d); RAIN (cont’d); thinking vs knowing; (un)resistance**

Reinforces noting practice and continues to train and support self-kindness**.** Specifically addresses the difference between trying to think our way out of uncertainty (or anxiety), and resting in a kind curious awareness of it. Modules also focus on not resisting experience and not getting tripped up by worry thinking.

**Modules 22-30: noting practice (cont’d); RAIN (cont’d); working with uncertainty and change**

Help individuals reflect on their own “evidence base” for working with worry to solidify their shift from reactivity to mindfully being with emotions as a new habit.

**Modules 30+: Reinforcing concepts via “theme weeks” + individual customization via “personal week”**

8 or more themed weeks and unlimited personalization of content by picking modules to develop a custom “week” for review.

### Outcome Measures

Participants completed self-administered surveys at three different time points: baseline, one month (primary endpoint), and three months (secondary endpoint) after treatment initiation. Data related to age and program engagement, as measured by the number of modules completed, was obtained directly from the UA app. Each survey contained the following measures.

#### Generalized Anxiety Disorder-7

The Generalized Anxiety Disorder-7 (GAD-7) is a 7-item questionnaire that is clinically used for measuring and tracking anxiety severity [38]. A 4-point Likert scale of frequency ranging from “not at all” to “nearly every day” is used to measure each item.

#### Maslach Burnout Inventory

Two single-item measures of emotional exhaustion and cynicism (originally called depersonalization) were included from the Maslach Burnout Inventory (MBI)-based on West’s [39] research, which demonstrated that these measures provided information on likelihood of burnout among medical professionals equivalent to the complete 22-item questionnaire (single emotional exhaustion question, *r_s_*=0.76-0.83; single cynicism question, *r_s_*=0.61-0.72) [40]. Furthermore, West [41] confirmed the concurrent validity of the single items in relation to the complete MBI in a follow-up study with physicians in 2011. These were measured using a 7-point Likert scale of frequency that ranged from “never” to “every day”.

#### Participant Satisfaction

Participants were asked their likelihood to recommend the program to a friend on an 11-point Likert scale that ranged from “not at all likely” to “extremely likely”.

### Statistical Analysis

The data was analyzed using R version 3.4.1 (R Foundation for Statistical Computing, Vienna, Austria). Friedman’s analysis of variance (ANOVA), a nonparametric test, was used to analyze the overall change in GAD-7 scores (primary outcome) and the single-item MBI scores (secondary outcomes) at the three time points due to the data having a nonnormal distribution. Post hoc analyses between the individual time points were analyzed using Wilcoxon signed-rank test and were corrected for multiple comparisons (Bonferroni). The relationship between GAD-7 and the single-item MBI scores was evaluated using Spearman’s correlation coefficient. Effect sizes were determined by dividing the z-score by the square root of the sample size to find Pearson’s *r* [42]. This uses Cohen’s criteria for *r* where 0.1 is a small effect, 0.3 is a medium effect, and 0.5 is a large effect [43].

## Results

### Profile of Participants

**Figure 2:**
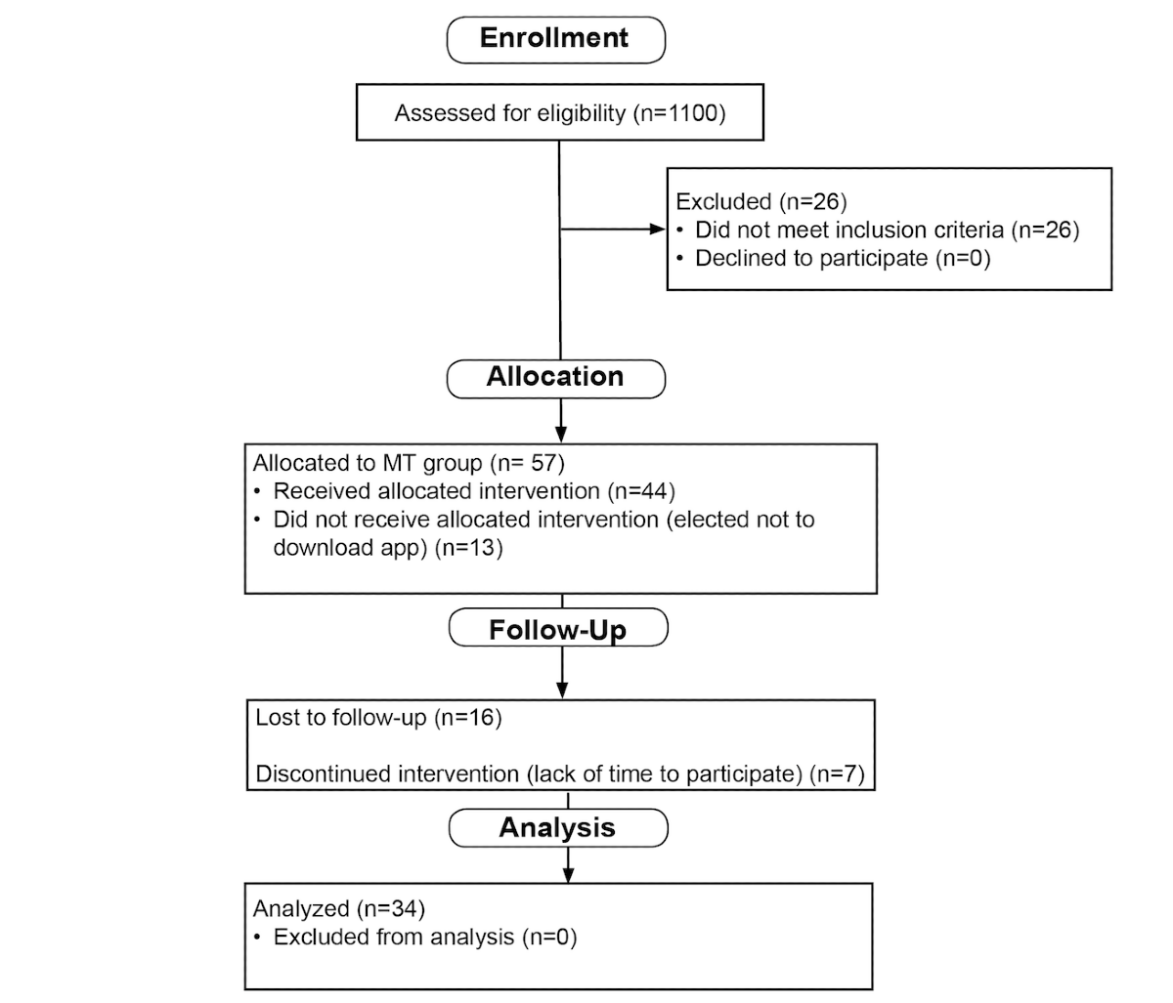
Consolidated Standards of Reporting Trials diagram. MT: mindfulness training.

A total of 57 participants met eligibility criteria and consented to participate during the spring and summer of 2018. Out of this group, 44 registered and downloaded the MT app. Out of 57 physicians, 34 (60%) were included in the final analysis (see Consolidated Standards of Reporting Trials diagram, Figure 2). The participant population was comprised of 25 women and 9 men who worked in health care for an average of 18 years (SD 10.25). The average age was 45 years (SD 8.89); although age data was not obtained for two individuals who completed the online surveys but did not download the smartphone app.

### Correlations Between Anxiety and Burnout

There was a significant correlation between anxiety and burnout at baseline, 1 month, and 3 months (Table 1) posttreatment initiation.

**Table 1:** Correlations between anxiety and burnout scores at baseline, 1 month, and 3 months after initiating mindfulness training.

Variables	GAD-7^a^ x emotional exhaustion	*P* value	GAD-7 x cynicism	*P* value
Baseline	0.71	<.001	0.43	.01
1 month	0.67	<.001	0.53	.001
3 months	0.53	.001	0.55	<.001

^a^GAD-7: Generalized Anxiety Disorder-7.

### Changes in Anxiety and Burnout

Friedman’s ANOVA showed there were significant changes across the three time points for GAD-7 (x^2^_2_=40.14, *P*<.001), emotional exhaustion (x^2^_2_=16.70, *P*<.001), and cynicism (x^2^_2_=10.13, *P*=.006).

**Figure 3:**
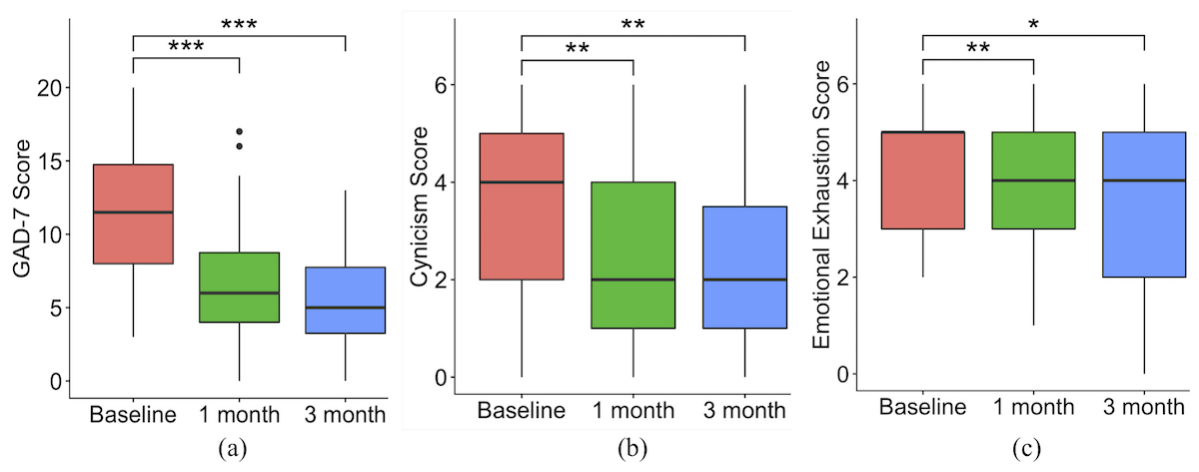
Box and whisker plots at baseline, one month, and three months for: (a) Generalized Anxiety Disorder-7 scores; (b) cynicism scores from Maslach Burnout Inventory; and (c) emotional exhaustion scores from Maslach Burnout Inventory. Significance level is denoted by asterisks: *=.05; **=.01; ***=.001. GAD-7: Generalized Anxiety Disorder-7.

Figure 3 shows box and whisker plots for each time point; the horizontal line inside the box indicates the median while the top of the box indicates the third quartile, and the bottom represents the first quartile. The whiskers indicate the maximum and minimum values and the dots represent outliers. Participants demonstrated a 48% reduction in GAD-7 scores from baseline (median 11.50, interquartile range [IQR] 8-14.75) to 1 month (median 6, IQR 4-8.75, *P*<.001, effect size 0.81; see Figure 3a) and a 57% reduction at 3 months (median 5, IQR 3.25-7.75, *P*<.001, effect size 0.85).

Participants reported a 50% reduction in cynicism scores from baseline (median 4, IQR 2-5) to 1 month (median 2, IQR 1-4, *P*=.003, effect size 0.54, Figure 3b) and a 50% reduction at 3 months (median 2, IQR 1-3.75, *P*=.009, effect size 0.49).

Participants reported a 20% reduction in emotional exhaustion scores from baseline (median 5, IQR 3-5) to 1 month (median 4, IQR 3-5, *P*<.001, effect size 0.63, Figure 3c) and a 20% reduction at 3 months (median 4, IQR 2-5, *P*=.003, effect size 0.56).

### Participant Engagement and Satisfaction

At 1 month, participants had completed an average of 11.03 (SD 9.51) modules of the 30-module program, and the average rating out of 10 for their likelihood to recommend the app to a friend was 7 (SD 2.79). At 3 months, participants had completed an average of 15.29 (SD 12.42) modules, and the average score for recommending the app was 8 out of 10 (SD 2.53).

## Discussion

This is the first study to directly test an app-delivered mindfulness-based treatment in anxious physicians and to assess the relationship between physician anxiety and burnout. We found preliminary evidence that app-based MT reduced both anxiety and burnout, demonstrating that this modality and type of training may be an accessible and possibly effective tool for helping busy physicians manage these conditions. We found significant correlations between anxiety and burnout at all time points, confirming the hypothesized link between the two. This is promising, as the majority of participants were midcareer and already experiencing anxiety and burnout. It is important to develop evidence-based tools to support these individuals in combatting burnout, as they are likely to spend at least an additional 15 years in the workforce. This work expands upon previous findings that a correlation exists between the anxiety, as measured by the State-Trait Anxiety Inventory, and burnout, as measured by the MBI, in health care workers in the 1990s [10]. Additionally, this has also been shown in Chinese physicians using the Zung Self-Rating Anxiety scale and the Chinese version of the MBI [44].

This study builds on previous research that indicates that mindfulness-based interventions delivered in-person can decrease anxiety in individuals with moderate to severe anxiety (eg, generalized anxiety disorder) and decrease physician burnout [20,28,45,46]. A large and growing number of app-based mindfulness and meditation trainings have emerged as treatment modalities for anxiety, yet few are theory-driven and even fewer are supported by clinical trials. As the field of digital therapeutics grows, it will be critical for new treatments to demonstrate mechanistic, empirical, and clinically meaningful effects. This study is unique in that it is the first to use app-based MT to directly target a theorized mechanism underlying anxiety (reinforcement learning) and link it to clinical outcomes (anxiety and burnout). These results demonstrate a proof-of-concept that app-based MT may be a promising modality for the treatment of anxiety in physicians and, perhaps, more broadly; however, carefully-designed randomized controlled studies in specific treatment realms are required as next steps.

The link between anxiety and burnout may not be surprising at face value, but it is important to directly establish this correlation in physicians from both a scientific and treatment standpoint, as it informs both observations and outcomes. Specifically for this study, MT was directed toward reinforcement learning pathways that perpetuate anxiety. No modules or even references of burnout, cynicism, or emotional exhaustion were made in the MT program. We theorized that if anxiety and burnout were correlated, we might see a reduction in both symptoms by singularly addressing anxiety. This was indeed the case, and provides further explanatory power as to why MT might reduce burnout in anxious physicians, given the strong correlations that were found between the two.

This study has several novel and notable findings; however, there are a number of limitations, including a relatively small and self-selected sample, a limited geographic area, and a single intervention arm. Proof-of-concept studies such as this one are a critical first step in exploring potential mechanisms and generating effect size calculations for larger randomized controlled trials. This study demonstrated that anxious physicians were willing to try an app-based MT program and might benefit from it. Another limitation is that the program was not tailored to physicians; it was designed to help anyone with moderate to severe anxiety.

MT has shown promise in helping physicians reduce burnout. This study shows a strong link between anxiety and some personal aspects of burnout, such as cynicism, and that targeting anxiety may help reduce both. This study also suggests that app-based MT may have promise as an accessible, evidence-based tool to combat anxiety and correlated aspects of burnout in physicians; although causal claims cannot definitively be made until future randomized controlled studies are conducted.

## Abbreviations

ANOVA: analysis of variance
GAD-7: Generalized Anxiety Disorder-7
IQR: interquartile range
MBI: Maslach Burnout Inventory
MBSR: mindfulness-based stress reduction
MT: mindfulness training
UA: Unwinding Anxiety.
